# Visual and non‐visual properties of filters manipulating short‐wavelength light

**DOI:** 10.1111/opo.12648

**Published:** 2019-11-06

**Authors:** Manuel Spitschan, Rafael Lazar, Christian Cajochen

**Affiliations:** ^1^ Department of Experimental Psychology University of Oxford Oxford UK; ^2^ Centre for Chronobiology Psychiatric Hospital of the University of Basel (UPK) Basel Switzerland; ^3^ Transfaculty Research Platform Molecular and Cognitive Neurosciences University of Basel Basel Switzerland

**Keywords:** blue‐blocking filters, circadian, ipRGCs, optics, short-wavelength light, sleep

## Abstract

**Purpose:**

Optical filters and tints manipulating short‐wavelength light (sometimes called ‘blue‐blocking’ or ‘blue‐attenuating’ filters) are used in the management of a range of ocular, retinal, neurological and psychiatric disorders. In many cases, the only available quantification of the optical effects of a given optical filter is the spectral transmittance, which specifies the amount of light transmitted as a function of wavelength.

**Methods:**

We propose a novel physiologically relevant and retinally referenced framework for quantifying the visual and non‐visual effects of these filters, incorporating the attenuation of luminance (*luminous transmittance*), the attenuation of melanopsin activation (*melanopsin transmittance*), the *colour shift*, and the reduction of the colour gamut (*gamut reduction*). Using these criteria, we examined a novel database of spectral transmittance functions of optical filters (*n* = 121) which were digitally extracted from a variety of sources.

**Results:**

We find a large diversity in the alteration of visual and non‐visual properties. The spectral transmittance properties of the examined filters vary widely, in terms of shapes and cut‐off wavelengths. All filters show relatively more melanopsin attenuation than luminance attenuation (lower melanopsin transmittance than luminous transmittance). Across the data set, we find that melanopsin transmittance and luminous transmittance are correlated.

**Conclusions:**

We suggest that future studies and examinations of the physiological effects of optical filters quantify the visual and non‐visual effects of the filters beyond the spectral transmittance, which will eventually aid in developing a mechanistic understanding of how different filters affect physiology. We strongly discourage comparing the downstream effects of different filters on, e.g. sleep or circadian responses, without considering their effects on the retinal stimulus.

## Introduction

### Background

Optical filters can be used to modify the visual input by blocking or attenuating light at specific parts of the visible spectrum.^1^, ^2^ So‐called ‘blue‐blocking’ or ‘blue‐attenuating’ filters reduce the amount of short‐wavelength light at the eye’s surface, the cornea. Optically, the filtering is typically realised using one of two ways: (1) using a cut‐off filter, which blocks or attenuates light below a specific wavelength, (2) using a notch filter, which blocks or attenuates light within a specific and limited short‐wavelength range, or a combination of both.

By altering the spectral distribution of the light incident on the retinal surface relative to no filtering,^3^ filters directly affect the activation of the cones and rods in the retina, which allow us to see during the day and night, respectively. In addition, the activity of the melanopsin‐containing intrinsically photosensitive retinal ganglion cells (ipRGCs) is also modulated by the use of optical filters. These cells, though only making up <1% of retinal ganglion cells, are of great importance to non‐visual functions, such as entrainment of circadian rhythms in physiology and behaviour to the environmental light‐dark cycle, and the suppression of melatonin in response to light.^4^

Previous investigations have examined the effects of filters manipulating short‐wavelength light on visual performance,^5^, ^6^, ^7^, ^8^, ^9^, ^10^, ^11^, ^12^, ^13^ colour vision,^11^, ^13^, ^14^, ^15^ and steady‐state and dynamic parameters of pupil size.^16^, ^17^ Furthermore, they have received attention in the domain of sleep medicine and chronobiology, where their effects on melatonin suppression,^18^, ^19^, ^20^, ^21^, ^22^, ^23^, ^24^, ^25^ circadian rhythms,^26^, ^27^, ^28^, ^29^, ^30^ sleep,^6^, ^17^, ^21^, ^25^, ^29^, ^30^, ^31^, ^32^, ^33^ modulation of alertness by light,^34^, ^35^ and for use by shift or night workers^36^, ^37^ have been investigated.

In addition, filters manipulating short‐wavelength light have been employed in the management of psychiatric and neurological (bipolar disorder,^38^, ^39^, ^40^ depression,^32^ ADHD,^41^ blepharospasm,^42^, ^43^ migraine,^44^, ^45^, ^46^ photosensitive epilepsy,^47^ insomnia^32^, ^41^, ^48^) and retinal conditions (rod monochromacy/achromatopsia,^49^, ^50^, ^51^, ^52^ retinitis pigmentosa,^53^ others 54), as well as for reducing non‐specific photophobia^46^, ^55^ and eye fatigue.^56^, ^57^ Coloured filters have also been used previously to improve reading difficulties and relieve visual stress^58^, ^59^ and symptoms of migraine,^60^ though these are not specifically attenuating short‐wavelength light^61^ (but may do so, depending on the specific filter chosen).

Across these studies and reports, filters by different manufacturers were used. In addition, the terms ‘blue‐blocking’ or ‘blue‐attenuating’, which are sometimes used to promote filters manipulating short‐wavelength light, are not well‐defined nor standardised. Therefore, there is great ambiguity about the exact properties of filters carrying those names.

The unique characteristic of a filter is its spectral transmittance, which is a quantified specification of how much light is passed through the filter at a given wavelength. Relating the transmittance properties of a filter to a specific retinal mechanism is a special case, e.g. in rod monochromacy, which specifically reduces the scotopic luminance. It is impossible to determine the precise effects on various visual and non‐visual functions just by examining the spectral transmittance function of a filter. While the transmittance is necessary to determine these effects quantitatively, this cannot be done ‘by eye’. To remedy this, we propose a retinally referenced framework for quantifying the visual effects.

### Retinal consequences of optical filters

**Figure 1:**
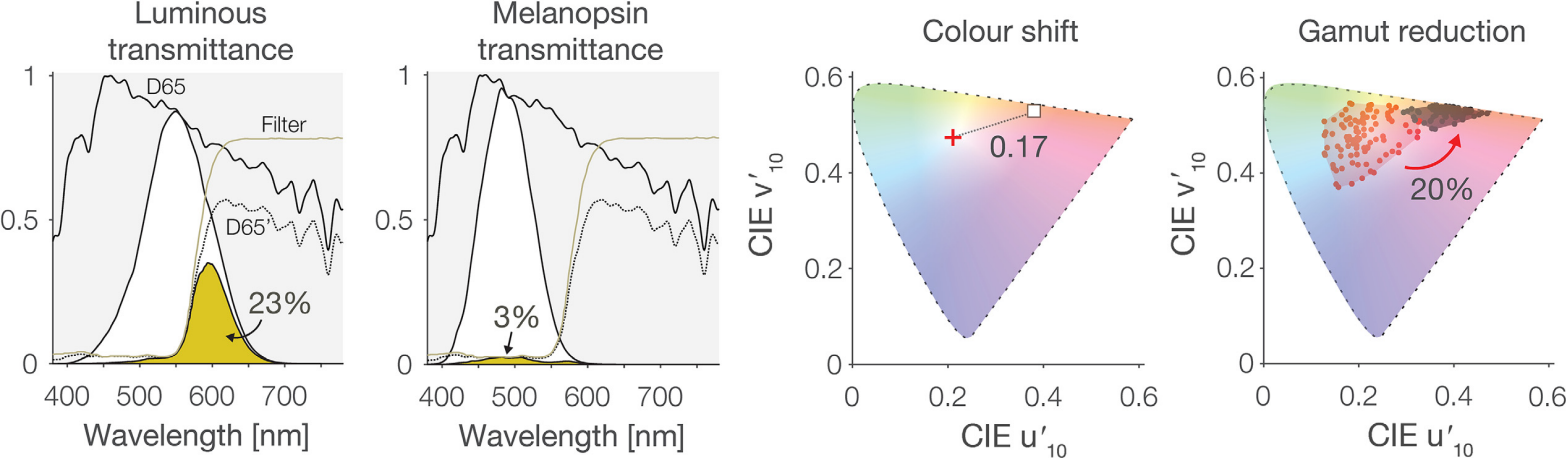
Visual and non‐visual effects of filters. *Luminous transmittance* is given as the *fraction of the* luminance of daylight seen with filter (shown as the beige area) to without filter (shown as the white area). In this case, the luminance with the filter is only about a quarter (23%)*.* Melanopsin *transmittance* follows the same calculation, except for the melanopsin photopigment. Because the cut‐off wavelength of the filter is outside of the spectral sensitivity of melanopsin, the attenuation is far larger compared to luminance (3%). Colour shift is the Euclidian distance *between a specified white point seen with and without the filter* in the uniform colour space used here (CIE 1976 u’_10_v’_10_ colour space based on the CIE 1964 10° observer.). The smaller the number, the smaller the colour shift *and therefore, the closer the reproduction of the white point with the filter relative to without the filter*. Gamut *reduction is the* reduction of the colour space which common surface reflectances inhabit, with and without the filter. In this case, the colour gamut with the filter is only around one fifth (20%) of the gamut without the filter. *Here and elsewhere,* the spectral power distribution was assumed to be of noon daylight (D65, daylight with a correlated colour temperature [CCT] of 6500K).

Changing the spectral distribution of the transmitted light can have a range of different effects. Here, we consider four main effects in the trichromatic human retina (*Figure* 1), all of which are important to assess the properties of a given filter:

1. *Luminous transmittance* [%]: By changing the spectral properties of the incident light, the luminance of the stimulus is altered. Luminance is calculated using the V(λ) curve, which is a combination of L and M cones. Luminous transmittance is the fraction of the luminance of the filtered spectral distribution relative to the luminance of the unfiltered spectral distribution. A luminous transmittance of 100% corresponds to no change in luminance by the filter. Values >100% are not possible.
2. *Melanopsin transmittance* [%]: The activation of melanopsin may be reduced by the optical filter under investigation. Using the same calculation procedure as for luminous transmittance, melanopsin transmittance is calculated as the proportion of melanopsin activation with filter relative to without filter.
3. *Colour shift:* Optical filters with non‐uniform transmittance also lead to a shift in the colour appearance of a colour which would appear as white without filter. With filters manipulating short‐wavelength light, these shifts typically occur in the yellow, orange or amber directions.
4. *Gamut reduction* [%]: Objects of different colours will appear different when seen with a filter. Without the filter, the distribution of differently coloured objects is called the gamut, which is simply the area which the object colours ‘inhabit’ in a colour space. We can compare the area of the gamut when objects are seen through a filter with the area of the gamut without the filter.

We note that these four properties of filters are by no means exhaustive, but they allow for a strongly quantifiable and yet intuitive approach of the effects of different optical filters on visual and non‐visual function. We also note that our analysis does not include consideration of transmission of light in the UV band and makes no claims about damaging effects of UV or other radiation. Similarly, we did not consider the polarisation properties of the filters.

### Parametric simulation of optical filters

**Figure 2:**
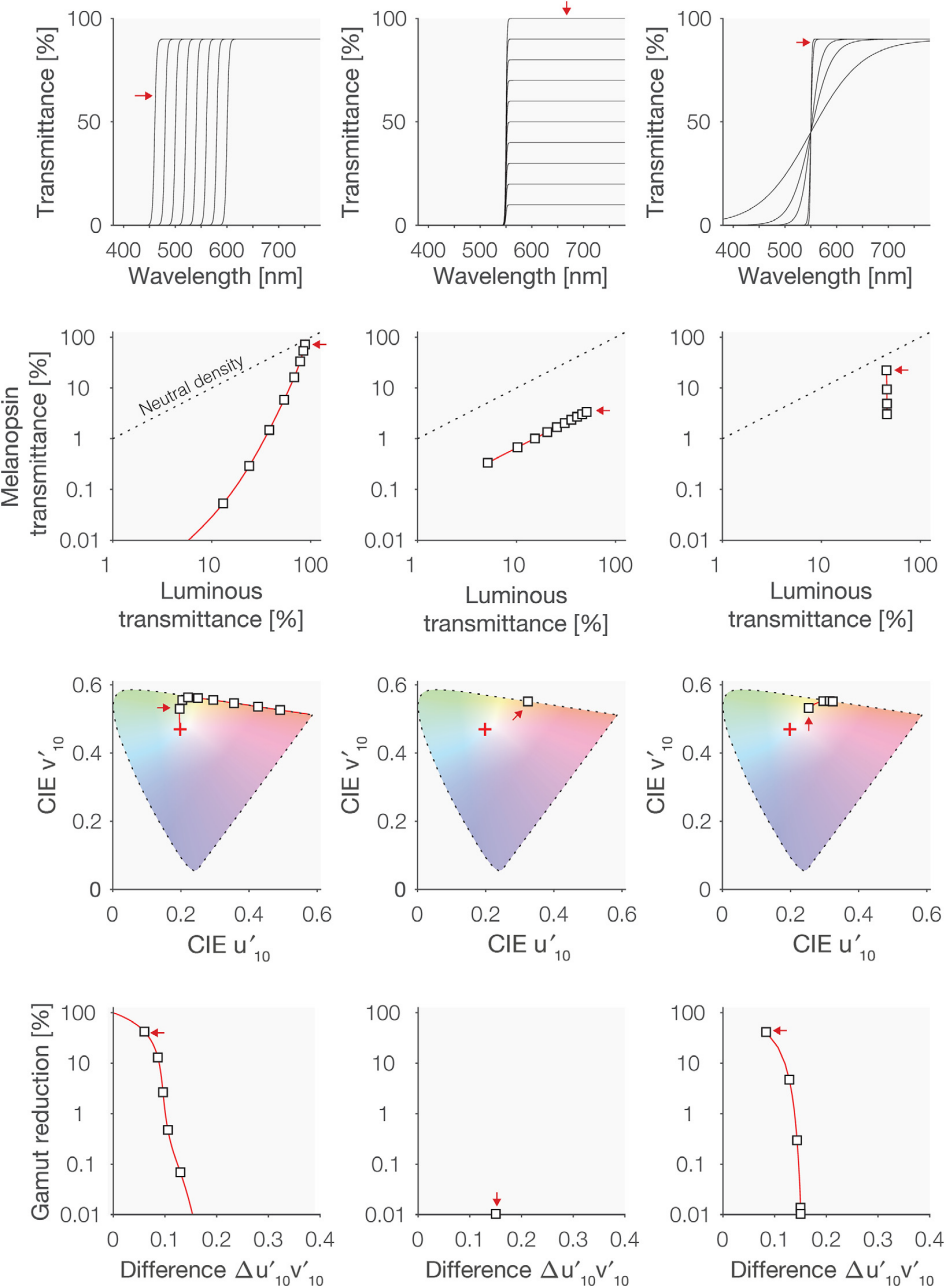
Exploring effects of cut‐off filters. Spectral transmittance of cut‐off filters is analytically modelled using the sigmoid function of the form $T\left(\lambda \right)=\frac{L}{1+e^{-k(\lambda -\lambda_{0})}}$, where L is the upper asymptote, k is the slope, and λ_0_ is the cut‐off wavelength. *Left column:* Varying cut‐off wavelengths (λ_0_), with L = 0.9, k = 0.5. *Middle column:* Varying top asymptote L, with λ_0_ = 550 nm, k = 0.5. *Right column:* Varying slope k, with λ_0_ = 560 nm, L = 0.9. Small arrows point to the spectral transmittances at one extreme end of specific parameter choice, pointing out the corresponding retinal effect in the other panels as well.

We started examining the visual and non‐visual filters using simulated filters. We used an analytic description of the typical transmittance profile using a sigmoid function (*Figure* 2). The model parameters were the (1) cut‐off wavelength, (2) the upper asymptote, and (3) the slope of the function. By changing each of these parameters in isolation, we can examine how properties of the spectral transmittance functions affect the derived parameters.

Varying the cut‐off wavelength systematically affects all four parameters (*Figure* 2, left column). Moving the cut‐off wavelength to longer wavelengths, leads to both more luminance attenuation and more melanopsin attenuation. However, because melanopsin spectral sensitivity has its peak at shorter wavelengths than the luminosity function, the increase in melanopsin attenuation happens at a faster rate than the increase in luminance attenuation. In the colour domain, cut‐off filters move the chromaticity towards the spectral locus, with larger deviations with longer cut‐off wavelengths.

At a fixed wavelength and slope, varying the asymptote, i.e. the ‘plateau’ and maximum transition systematically effects only luminance and melanopsin, though to the same amount (*Figure* 2, middle column), producing a line that is parallel to the neutral density locus as one would expect. The chromaticity of filters with varying plateaus is the same. The slope of the transmittance function (*Figure* 2, right column) at a fixed cut‐off wavelength affects largely the attenuation of melanopsin and the change in colour properties, but not so much luminance, though we expect that this will depend on the cut‐off wavelength itself.

### Novel database of optical filters

In this work, we examined the spectral transmittance functions of 121 filters manipulating short‐wavelength light (either for spectacles or for contact lenses) with respect to their luminous transmittance, melanopsin transmittance, colour shift, and gamut reduction. These filters were obtained using digital data extraction techniques from a variety of sources, including published graphs from scientific articles, informative patient brochures and manufacturer brochures.

We classify these filters in three loose yet intuitive ad‐hoc categories: medical filters (*n* = 76), safety filters (*n* = 11) and task‐specific filters (*n* = 34). This latter category is subdivided into filters for sports (*n* = 10), driving (*n* = 4), visual display unit (VDU) use (*n* = 12) and a catch‐all ‘Other’ category (*n* = 8). In this analysis, we are agnostic to the materials that these filters are applied on or produced from, though this is an important consideration for practical use. All extracted filter transmittances are available on the open‐access repository.

## Methods

### Data sourcing and extraction

Published graphs of the spectral transmittance of filters manipulating short‐wavelength light were sourced using informal searches on PubMed, Google Scholar, and Google, yielding a total of 121 filters to be considered. Where data were obtained from manufacturers’ or other websites, those websites were submitted to the Internet Archive Wayback Machine for permanent archiving (http://www.web.archive.org/).

Transmittance spectra were extracted by one operator (author of this study, RL) from published graphs using WebPlotDigitzer 4.1 (http://automeris.io/WebPlotDigitizer),^62^ a free visual tool for extraction of data points from published graphs where no tabulated data are available. WebPlotDigitizer has been found to provide rather high levels of reliability.^63^ Axis calibration and data points in the given curves were set as precisely as visual inspection allowed. The maximum range of values clearly identifiable in the graphs were used for scaling. All transmission data extraction files underwent a secondary inspection. In some cases where multiple transmission curves were given in one plot, curves overlapped. If in these cases some of the transmission curves were overdrawn and thus not clearly visible, we assumed that the ‘top’ curve also corresponded to the other overdrawn curves. After extraction, data were then interpolated to 1 nm resolution using piecewise cubic hermite interpolating polynomial (PCHIP) interpolation. At wavelengths at the short‐wavelength and long‐wavelength extreme of the visible range at which in some cases no data were available, the transmittance was set to 0, which has the effect that light at those wavelengths is not factored into the calculation. If negative transmission values occurred, the data extraction was again checked and validated. The remaining negative values due to inaccuracy in the given curves were then set to 0.

### Calculation procedures

For calculation of melanopsin activation, we used the spectral sensitivity recently standardised by the Commission Internationale de l’Eclairage as CIE S 026/E:2018,^64^ which assumes a Govardovskii nomogram (λ_max_ = 480 nm) as well as a custom lens function synthesizing different lens transmittance functions.^4^ While the standard also recommends functions for the cones,^65^ we opted for the CIE 1964 10° observer in this work to maximise compatibility of the colorimetric calculations. This observer prescribes XYZ functions which are converted to the well‐known u’_10_v’_10_ space.

Luminous transmittance was calculated as the fraction of the luminance of the light seen with or without the filter incorporated using numeric integration: ${\text{Transmittance}}_{\text{Luminance}}=\frac{\sum_{380}^{780}F\left(\lambda \right)E_{D65}\left(\lambda \right)V_{10^{\circ }}\left(\lambda \right)\Delta \lambda }{\sum_{380}^{780}E_{D65}\left(\lambda \right)V_{10^{\circ }}\left(\lambda \right)\Delta \lambda }$, where *F*(λ) corresponds to the spectral transmittance of the filter, *E*_D65_(λ) to the spectrum of daylight at 6500K (D65), and *V*_10°_ (λ) to the CIE 1964 10° luminosity function.

Melanopsin transmittance was calculated using the same procedure, except with the melanopsin spectral sensitivity: ${\text{Transmittance}}_{\text{Melanopsin}}=\frac{\sum_{380}^{780}F\left(\lambda \right)E_{D65}\left(\lambda \right)S_{\text{Melanopsin}}\left(\lambda \right)\Delta \lambda }{\sum_{380}^{780}E_{D65}\left(\lambda \right)S_{\text{Melanopsin}}\left(\lambda \right)\Delta \lambda }$, where *F*(λ) corresponds to the spectral transmittance of the filter, E_D65_(λ) to the spectrum of daylight at 6500 K (D65), and *S*_Melanopsin_ (λ) to the CIE melanopsin spectral sensitivity function.

The colour shift was calculated as the Euclidean distance between the chromaticities of the D65 illuminant seen with and without the filter in the uniform CIE 1976 u’_10_v’_10_ colour space based on the CIE 1964 10° observer. The colour gamut with and without the filter was calculated using the IES Color Evaluation Samples (CES), a set of 99 representative reflectances^66^ selected from a large set of 105 000 spectral reflectance functions of paints, textiles, natural objects, skin, tones, inks, and other functions. Gamut reduction was calculated using the ratio of the area of the convex hulls of these 99 reflectance functions seen with and without the filter. The different effects are visualised in *Figure* 1.

### Data and software availability

**Figure 3:**
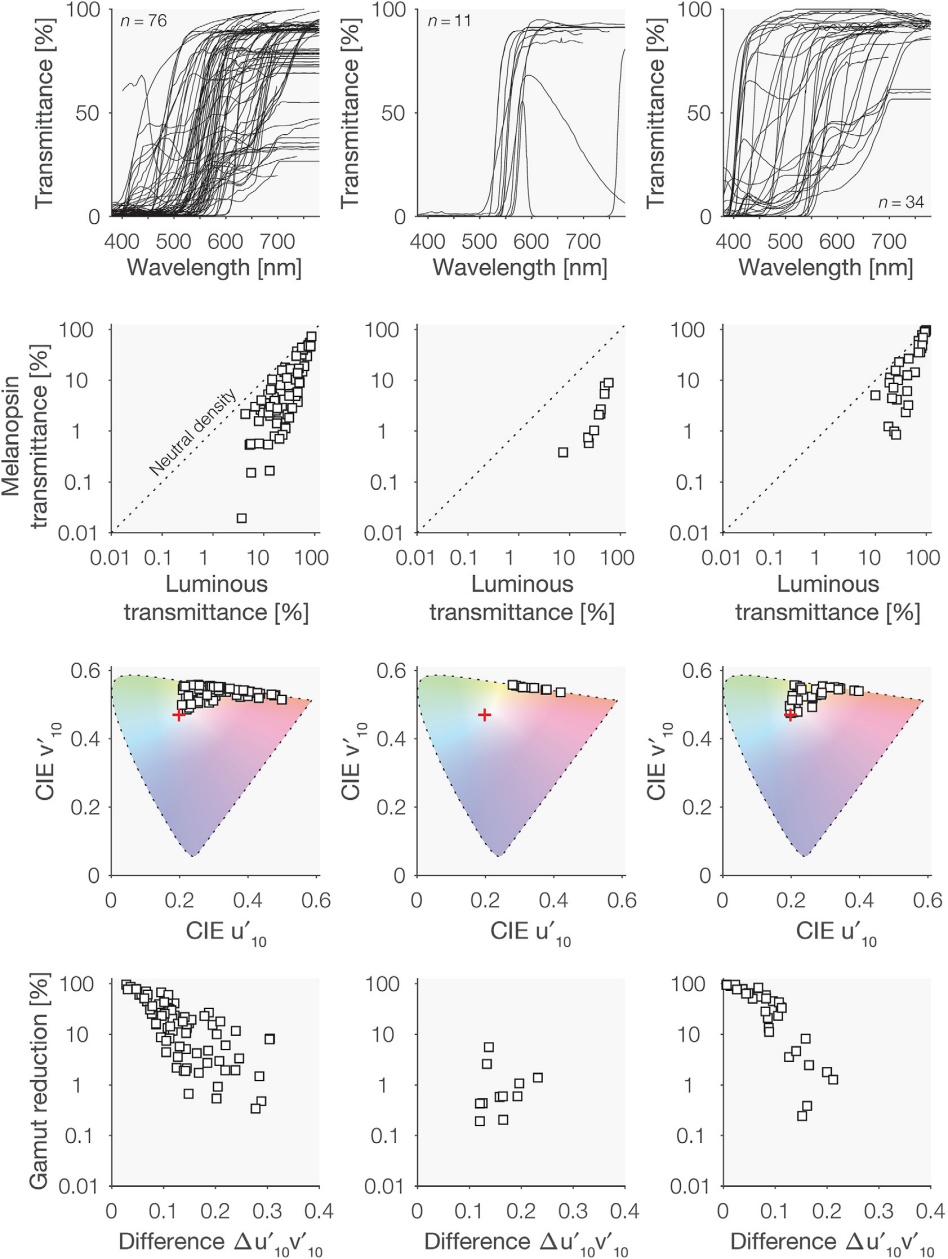
Visual and non‐visual properties of *spectral* filters. Columns correspond to the three main filter categories we identified (*Figure* 2). *Row 1*: Spectral transmittances of filters. *Row 2*: Luminous transmittance vs melanopsin transmittance. Dashed line indicates equal reduction of luminance and melanopsin, as would be the case with a spectrally uniform neutral density (ND) filter. *Row 3*: Chromaticity diagram. The red cross indicates chromaticity of 6500K daylight (D65) and white squares indicate chromaticities of D65 seen through the respective filters. *Row 4*: Colour shift vs gamut factor. See Introduction and *Figure* 1 for explanation.

All tabulated transmittance spectra and code to produce the unedited versions in the figures in this report are available on GitHub (https://github.com/spitschan/Spitschan2019_OPO). *Table* S1 contains the list of all filters, along with the four tabulated effects. *Data* S1 contains the data shown in *Figure* 3 and given in *Table* S1. *Data* S2 contains the transmittance spectra.

## Results

### Large diversity of filters

Across all categories, the spectral transmittances of our spectral filters (*n* = 121, see *Table* S1 for description of sources) are rather diverse (*Figure* 3, row 1). Firstly, this diversity is reflected in the shape of the filter transmittance function, i.e. whether it is a cut‐off, notch or some other form of filter. Furthermore, the cut‐off filters differ largely in the wavelength at which they transmit 50% of light. Another differing factor is the ‘plateau’ of transmission at wavelengths longer than the cut‐off wavelength.

All filters show more relative melanopsin attenuation than luminance attenuation (*Figure* 3 *,* row 2). This is evidenced by the fact that all data points lie under the identity line, which corresponds to a spectrally flat filter which decreases the activation of all photopigments by the same amount. It appears as though there is filters consistently inhabit a triangular segment in the luminance‐melanopsin attenuation space, bounded by the neutral density along the identity line at the upper end.

All filters show a move of the chromaticity of the D65 white point towards the spectral locus, which is given by the edge of the chromaticity diagram (*Figure* 3 *, row 3*). This locus corresponds to the chromaticity of monochromatic and therefore highly saturated lights. In the colour rendering properties of the filters quantified using the gamut attenuation, we find no clear pattern (*Figure* 3 *,* row 4*)*. We find that some filters severely reduce the colour gamut of the worlds seen with the filter, reducing it to only <1% of the gamut seen with the filter. This is obviously correlated with the shift of the filter towards the spectral locus as all surfaces seen under monochromatic illumination appear to only change in brightness, and not colour (because the surfaces can only reflect the light that is there). As would be expected, larger colour shifts (i.e. shift of the colour towards the spectral locus) translate to a reduced colour gamut.

Overall, we find that these response variables are correlated, though to different extents (*Table* 1). The largest correlation is between the luminous transmittance and the melanopsin transmittance. This result can be understood intuitively: Due to the overlap in the spectral sensitivities of luminance and melanopsin, it is practically impossible to modulate one without the other (see also *Figure* 2). Similarly, the larger a colour shift by a filter, the smaller the gamut: As a filter pushes the visual scene to the spectral locus (i.e. the edges of the chromaticity diagram), the less light reflected from surfaces in the visual scene will be transmitted.

**Table 1:** Correlation matrix between variables

	Melanopsin transmittance	Colour difference	Colour gamut
Luminous transmittance	0.86	−0.65	0.43
Melanopsin transmittance		−0.74	0.74
Colour difference			−0.78

## Discussion

### General discussion

In our analysis we find that there is large variability in the visual and non‐visual properties filters manipulating short‐wavelength light. While the spectral transmittance of a filter necessarily needs to be known for quantifying the four outcomes we investigated here, it in itself is not of any use as to determine a filter’s effect on the retina. In addition to providing transmittance spectra in tabulated form, future studies should consider quantifying the effect of a filter using the metrics described here.

For many of the applications of filters manipulating short‐wavelength light reaching the retina, we still lack a mechanistic understanding of how the different photoreceptors contribute to the effects. The precise mechanism which a given filter modifies is known only in special cases, e.g. in rod monochromacy.^51^ For example, at present (2019), we do not know how cones and rods contribute to the basic physiological regulation of melatonin secretion by light. A retinally referenced, or ‘physiologically relevant’ framework to quantify effects of a filter is the first step in using optical filters for developing such mechanistic understanding, which needs to be the basis for the development of recommendations for filter use for specific conditions.

### Pupil size effects

**Figure 4:**
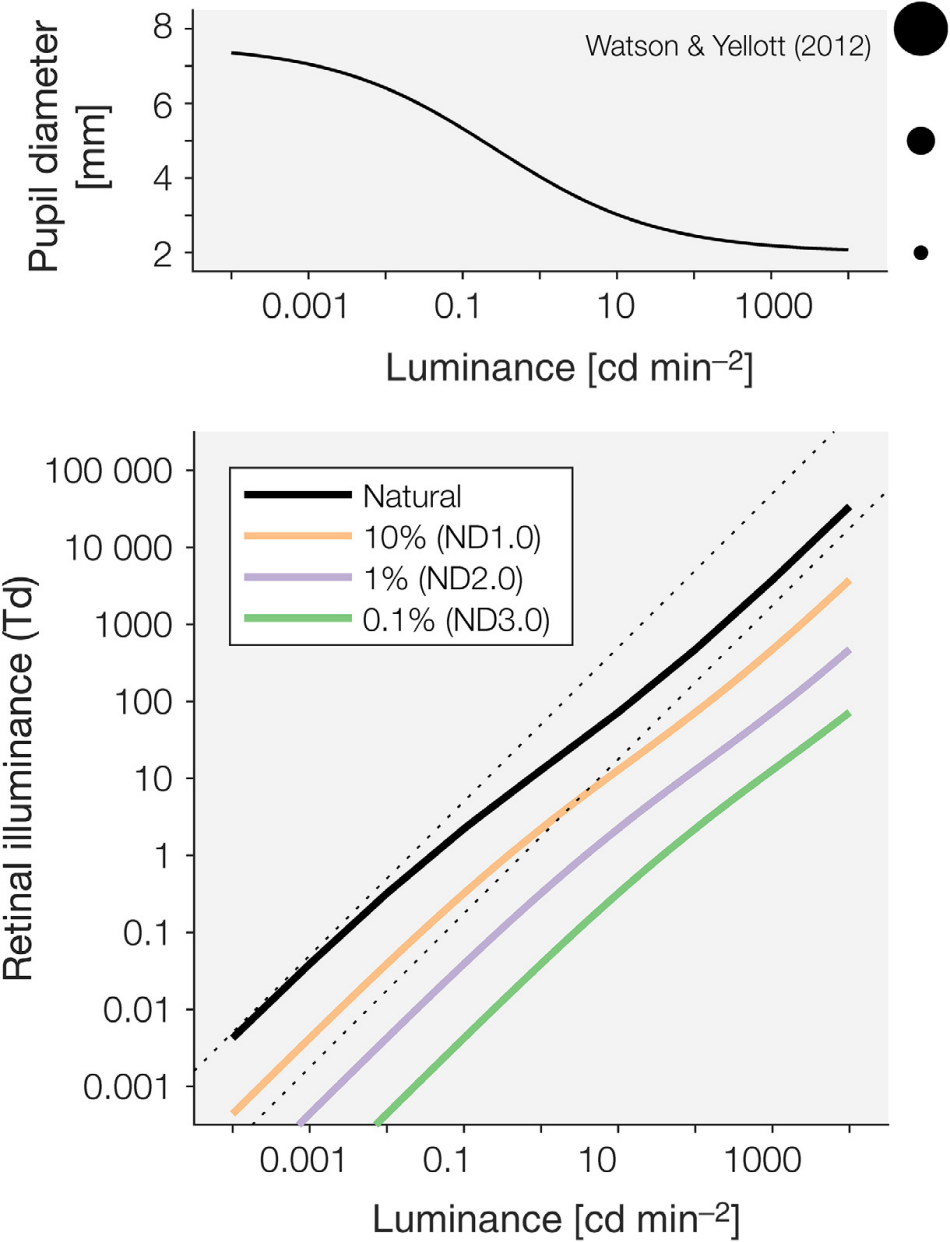
Pupil size effects of neutral‐density filters. *Top panel:* Predicted pupil size for a 32‐year old observer viewing a 150° field at varying luminances with both eyes. *Bottom panel:* Predicted retinal illuminance (luminance × pupil area) when viewed either through the natural pupil (dashed lines indicate maximum retinal illuminance given maximum difference in pupil size), or through spectrally uniform filters of varying transmittance (ND1.0, ND2.0, ND3.0).

The reduction of illumination at the cornea by optical filters reduces the retinal illuminance, i.e. the incident light on the retinal surface. However, retinal illuminance itself is also controlled by the area of the pupil. With a reduction in light intensity, the pupil area becomes bigger. The dynamic range, however, is rather limited, with a maximum possible modulation of retinal illuminance just by pupil size by a factor of ~16 (between a maximally constricted 2 mm pupil and a maximally dilated 8 mm pupil). We investigated the effect of reducing the corneal illuminance with optical filters on the effective retinal illuminance using the unified Watson & Yellott model^67^ (*Figure* 4). We assumed a field size of 150°, a 32‐year old observer, as well as binocular stimulation as an approximation to real world viewing conditions, and investigated how the resulting predicted retinal illuminance depends on the luminance of the viewed stimulus with and without neutral density filters of varying densities (ND1.0, ND2.0 and ND3.0). With the near‐parallel lines of log luminance vs log retinal illuminance, it can be seen that the primary determinant of retinal illuminance is luminance ‘seen’ through the filter.

Chung and Pease^16^ note that at equivalent luminance, ‘yellow’ filters lead to larger pupils than neutral‐density filters. This is consistent with the view that melanopsin activation, which significantly drives steady‐state pupil size in humans,^68^, ^69^ is severely reduced under short‐wavelength filters. Under most real‐world conditions, the correlation between luminance and melanopsin activation is probably well‐constrained, except with chromatic filters and experimental conditions in which the decoupling can be lead to up to a three‐fold difference in melanopsin activation with no or little nominal difference in luminance.^70^, ^71^ Importantly, optical quality of the retinal image depends on pupil size,^72^ which needs to be factored into filter assessments.

### Digital data extraction

This study relied on digital data extraction from published graphs. This was necessary because spectral transmittance data are rarely available in digital or tabulated form, even though data storage as supplementary material is often available at scientific journals at the time of writing this article (2019). As shown in this article, this need not be a limitation since data can be extracted from graphs and be subjected to rigorous novel or reanalyses. A limitation of our data‐driven approach is that tabulated spectra given by manufacturers are here treated as *bona fide* measurements of transmittance spectra. The process of data extraction using this method may also lead to inaccuracies in the tabulated transmittance spectra. These inaccuracies arise from the conversion of graphics (often low‐resolution) to numerical values. Inaccuracies in the assumed transmittances may lead to inaccuracies in the estimation of the effects of the filters.

### Other considerations

In some real‐world scenarios, the short‐wavelength properties of lighting or light‐emitted devices may be modified directly (e.g. using applications changing the colour balance on VDUs,^71^ or spectrally tuneable lighting^73^), but this is not the general case. Optical filters therefore represent a practical alternative for real‐world scenarios.

In addition to filters affecting the retinal stimulus, light of different colours may also have psychological or higher‐order effects.^74^ Another effect worth considering is stigma towards wearers of coloured spectacles.^75^ Here, we only considered the visual and non‐visual properties of different optical filters. Other optical effects such as scattering or polarisation and other higher‐order effects are not captured by the simple filter model that we have applied here.

The proposed retinally referenced framework does not take into consideration the role of adaptation to the modified spectral environments.^76^, ^77^, ^78^ In addition to adaptive mechanisms at play, there may be other long‐term changes in visual processing in habitual wearers of coloured filters.^79^ The degree of short‐term and long‐term adaptation in visual and non‐visual function in response to specific filter types is an interesting empirical question.

## Conclusion

We find large diversity in the visual and non‐visual properties of different spectral filters manipulating short‐wavelength light. We propose that to evaluate the effect of a given optical filter, the spectral transmittance is only the first step in characterising the effect of a filter on the illumination at the eye and suggest a retinally referenced framework to quantify these effects, incorporating the attenuation of luminance, the attenuation of melanopsin activation, shifts in colour, and reduction of colour gamut.

## Conflict of interest

The authors report no conflicts of interest and have no proprietary interest in any of the materials mentioned in this article.

## Supporting information


**Table S1.** List of filters.

**Data S1.** Visual and non‐visual properties of the examined filters.

**Data S2.** Spectral transmittances of the examined filters.
