# Supplementary material for: Visual and non-visual properties of filters manipulating short-wavelength light
Source: Ophthalmic Physiol Opt. 2019 Nov 6;39(6):459–68. doi: 10.1111/opo.12648 (PMC6887545; doi:10.1111/opo.12648)
Supplement: Supplementary file 1 — Table S1. List of filters. [file 44402_2019_3906008_MOESM1_ESM.docx]

**Supplementary Table**

**Table S1: List of filters**

| **Manufacturer** | **Product name** | **Reference** | Filter type | Luminous transmittance [%] | Melanopsin transmittance [%] | Colour difference | Gamut reduction [%] |
| --- | --- | --- | --- | --- | --- | --- | --- |
| 3M | 2846 red-orange | Technical fact sheet, from [website (archived)](https://web.archive.org/web/20190314184848/https:/static.rapidonline.com/pdf/527782_v1.pdf) | Safety lenses | 49.3 | 7.6 | 0.13 | 2.6 |
| Brain Power Inc. | DiamondDye 540 nm | BPI [1], [website](https://web.archive.org/web/20190314180807/https:/www.callbpi.com/pdf_misc/thera.pdf) | Medical | 18.1 | 1.4 | 0.16 | 4.2 |
| Brain Power Inc. | DiamondDye 550 nm | BPI [1], [website](https://web.archive.org/web/20190314180807/https:/www.callbpi.com/pdf_misc/thera.pdf) | Medical | 26.1 | 1.2 | 0.21 | 3 |
| Brain Power Inc. | Driver Tint | BPI [1], [website](https://web.archive.org/web/20190314180807/https:/www.callbpi.com/pdf_misc/thera.pdf) | Task-specific – Driving | 18 | 1.2 | 0.17 | 2.5 |
| Brain Power Inc. | Euro-Brown | BPI [1], [website](https://web.archive.org/web/20190314180807/https:/www.callbpi.com/pdf_misc/thera.pdf) | Medical | 13.7 | 6.5 | 0.09 | 39.6 |
| Brain Power Inc. | FL-41 Tint | BPI [1], [website](https://web.archive.org/web/20190314180807/https:/www.callbpi.com/pdf_misc/thera.pdf) | Medical | 28.4 | 11.1 | 0.11 | 60 |
| Brain Power Inc. | Golf Tint | BPI [1], [website](https://web.archive.org/web/20190314180807/https:/www.callbpi.com/pdf_misc/thera.pdf) | Task-specific – Sports | 29.3 | 22.8 | 0.04 | 78.4 |
| Brain Power Inc. | Monochrome 600 nm | Herz and Yen [2] | Medical | 5.2 | 0.5 | 0.3 | 8.3 |
| Brain Power Inc. | Ski Tint | BPI [1], [website](https://web.archive.org/web/20190314180807/https:/www.callbpi.com/pdf_misc/thera.pdf) | Task-specific – Sports | 18.8 | 9 | 0.11 | 34.3 |
| Brain Power Inc. | Sport Tint | BPI [1], [website](https://web.archive.org/web/20190314180807/https:/www.callbpi.com/pdf_misc/thera.pdf) | Task-specific – Sports | 25 | 15.6 | 0.08 | 51 |
| Brain Power Inc. | Tennis Tint | BPI [1], [website](https://web.archive.org/web/20190314180807/https:/www.callbpi.com/pdf_misc/thera.pdf) | Task-specific – Sports | 85.9 | 63.7 | 0.06 | 50.8 |
| Brain Power Inc. | Winter Sun | BPI [1], [website](https://web.archive.org/web/20190314180807/https:/www.callbpi.com/pdf_misc/thera.pdf) | Task-specific – Other | 80.1 | 42.8 | 0.09 | 13.4 |
| Chron-Optic inc | N/A - orange lens | van der Lely, Frey [3] | Task-specific – Other | 21.3 | 4.4 | 0.16 | 8.2 |
| Chron-Optic inc. | N/A - orange lens | Sasseville, Benhaberou-Brun [4] | Task-specific – Other | 42.2 | 6.1 | 0.14 | 4.7 |
| Chron-Optic inc. | N/A - orange lens | Sasseville and Hébert [5] | Task-specific – Other | 23.5 | 1 | 0.2 | 1.8 |
| Corning | Corning 511 | Plum and Gerull [6], [website](https://web.archive.org/web/20190314200110/http:/satis.de/links/05_downloads/Kantenfilter2008.pdf) | Medical | 42.5 | 10.3 | 0.11 | 14.4 |
| Corning | Corning 527 | Plum and Gerull [6], [website](https://web.archive.org/web/20190314200110/http:/satis.de/links/05_downloads/Kantenfilter2008.pdf) | Medical | 32.1 | 5.4 | 0.14 | 17.9 |
| Corning | Corning 550 | Plum and Gerull [6], [website](https://web.archive.org/web/20190314200110/http:/satis.de/links/05_downloads/Kantenfilter2008.pdf) | Medical | 18.5 | 2.8 | 0.18 | 23.1 |
| Corning | CPF550S | Schwerdtfeger and Gräf [7] | Medical | 18.5 | 1.6 | 0.2 | 10 |
| DAO-AG | 0C1 | [Website (archived)](https://web.archive.org/web/20190314190225/https:/www.dao-ag.de/kantenfilter.html) | Medical | 75.6 | 49.9 | 0.05 | 60.2 |
| DAO-AG | L400 | [Website (archived)](https://web.archive.org/web/20190314190225/https:/www.dao-ag.de/kantenfilter.html) | Task-specific – VDU use | 93.1 | 91.6 | 0.01 | 94.5 |
| DAO-AG | L41-A | [Website (archived)](https://web.archive.org/web/20190314190225/https:/www.dao-ag.de/kantenfilter.html) | Task-specific – VDU use | 82.6 | 77.5 | 0.01 | 95.2 |
| DAO-AG | L41-B | [Website (archived)](https://web.archive.org/web/20190314190225/https:/www.dao-ag.de/kantenfilter.html) | Task-specific – VDU use | 70.6 | 59.8 | 0.02 | 95.6 |
| DAO-AG | L41-C | [Website (archived)](https://web.archive.org/web/20190314190225/https:/www.dao-ag.de/kantenfilter.html) | Task-specific – VDU use | 45.1 | 26.6 | 0.07 | 83.1 |
| DAO-AG | L450 | [Website (archived)](https://web.archive.org/web/20190314190225/https:/www.dao-ag.de/kantenfilter.html) | Medical | 80.7 | 46.2 | 0.09 | 15.6 |
| DAO-AG | L480-50 | [Website (archived)](https://web.archive.org/web/20190314190225/https:/www.dao-ag.de/kantenfilter.html) | Medical | 44.9 | 36.9 | 0.03 | 79.5 |
| DAO-AG | L480-75 | [Website (archived)](https://web.archive.org/web/20190314190225/https:/www.dao-ag.de/kantenfilter.html) | Medical | 22.7 | 15.7 | 0.06 | 62.4 |
| DAO-AG | L480-90 | [Website (archived)](https://web.archive.org/web/20190314190225/https:/www.dao-ag.de/kantenfilter.html) | Medical | 6.8 | 3 | 0.1 | 25.7 |
| DAO-AG | L500 | [Website (archived)](https://web.archive.org/web/20190314190225/https:/www.dao-ag.de/kantenfilter.html) | Medical | 70 | 34 | 0.08 | 36.3 |
| DAO-AG | L500-H | [Website (archived)](https://web.archive.org/web/20190314190225/https:/www.dao-ag.de/kantenfilter.html) | Medical | 62.6 | 18.9 | 0.1 | 4.4 |
| DAO-AG | L511 | [Website (archived)](https://web.archive.org/web/20190314190225/https:/www.dao-ag.de/kantenfilter.html) | Medical | 49.7 | 8.9 | 0.13 | 3.6 |
| DAO-AG | L511-H | [Website (archived)](https://web.archive.org/web/20190314190225/https:/www.dao-ag.de/kantenfilter.html) | Medical | 50.7 | 8.9 | 0.12 | 2.2 |
| DAO-AG | L527 | [Website (archived)](https://web.archive.org/web/20190314190225/https:/www.dao-ag.de/kantenfilter.html) | Medical | 41.9 | 4.9 | 0.14 | 2.2 |
| DAO-AG | L527-H | [Website (archived)](https://web.archive.org/web/20190314190225/https:/www.dao-ag.de/kantenfilter.html) | Medical | 45.1 | 5.6 | 0.14 | 1.9 |
| DAO-AG | L540-50 | [Website (archived)](https://web.archive.org/web/20190314190225/https:/www.dao-ag.de/kantenfilter.html) | Medical | 48.7 | 17 | 0.1 | 23.3 |
| DAO-AG | L540-70 | [Website (archived)](https://web.archive.org/web/20190314190225/https:/www.dao-ag.de/kantenfilter.html) | Medical | 31.5 | 5 | 0.13 | 5.8 |
| DAO-AG | L550 | [Website (archived)](https://web.archive.org/web/20190314190225/https:/www.dao-ag.de/kantenfilter.html) | Medical | 31.5 | 1.8 | 0.18 | 2.7 |
| DAO-AG | L585 | [Website (archived)](https://web.archive.org/web/20190314190225/https:/www.dao-ag.de/kantenfilter.html) | Medical | 16.3 | 0.8 | 0.25 | 3.4 |
| DAO-AG | LC1-H | [Website (archived)](https://web.archive.org/web/20190314190225/https:/www.dao-ag.de/kantenfilter.html) | Medical | 71 | 29.7 | 0.09 | 8.8 |
| Eschenbach | 450 | Sasseville, Paquet [8] | Medical | 81.3 | 52.6 | 0.07 | 30.9 |
| Eschenbach | 511 | Plum and Gerull [6], [website](https://web.archive.org/web/20190314200110/http:/satis.de/links/05_downloads/Kantenfilter2008.pdf) | Medical | 57.4 | 14.8 | 0.11 | 8.3 |
| Eschenbach | 527 | Plum and Gerull [6], [website](https://web.archive.org/web/20190314200110/http:/satis.de/links/05_downloads/Kantenfilter2008.pdf) | Medical | 43 | 5.5 | 0.14 | 5.1 |
| Eschenbach | 550 | Plum and Gerull [6], [website](https://web.archive.org/web/20190314200110/http:/satis.de/links/05_downloads/Kantenfilter2008.pdf) | Medical | 29.3 | 2.1 | 0.19 | 4.7 |
| Eschenbach | 450 POL | Plum and Gerull [6], [website](https://web.archive.org/web/20190314200110/http:/satis.de/links/05_downloads/Kantenfilter2008.pdf) | Medical | 32.9 | 16.7 | 0.08 | 34.6 |
| Eschenbach | 511 POL | Plum and Gerull [6], [website](https://web.archive.org/web/20190314200110/http:/satis.de/links/05_downloads/Kantenfilter2008.pdf) | Medical | 13.5 | 3 | 0.14 | 22 |
| Eschenbach | 527 POL | Plum and Gerull [6], [website](https://web.archive.org/web/20190314200110/http:/satis.de/links/05_downloads/Kantenfilter2008.pdf) | Medical | 16.2 | 3.9 | 0.13 | 16.3 |
| Eschenbach | 550 POL | Plum and Gerull [6], [website](https://web.archive.org/web/20190314200110/http:/satis.de/links/05_downloads/Kantenfilter2008.pdf) | Medical | 12.3 | 2.3 | 0.15 | 19.3 |
| Eschenbach | Solar Shield Ultra | Krüger, Bullmann [9] | Medical | 23.3 | 0.8 | 0.2 | 0.5 |
| Eschenbach | Wellness PROTECT wp15 | Plum and Gerull [6], [website](https://web.archive.org/web/20190314200110/http:/satis.de/links/05_downloads/Kantenfilter2008.pdf) | Medical | 46.4 | 13.9 | 0.11 | 7.5 |
| Essilor | Crizal Prevencia | Lin, Gerratt [10] | Task-specific – VDU use | 96.8 | 90.9 | 0.01 | 90 |
| Essilor | Orma RT 50% | Plum and Gerull [6], [website](https://web.archive.org/web/20190314200110/http:/satis.de/links/05_downloads/Kantenfilter2008.pdf) | Medical | 26.1 | 17.9 | 0.06 | 66.6 |
| Essilor | Orma RT 85% | Plum and Gerull [6], [website](https://web.archive.org/web/20190314200110/http:/satis.de/links/05_downloads/Kantenfilter2008.pdf) | Medical | 12.1 | 5.3 | 0.11 | 49 |
| Hoggan et al 2015 | N/A - 480nm notch filter | Hoggan, Subhash [11] | Medical | 59.2 | 40 | 0.03 | 96.4 |
| Honeywell Uvex | SCT Orange | Ostrin, Abbott [12] | Safety lenses | 37.6 | 2.1 | 0.16 | 0.6 |
| Honeywell Uvex | SCT Orange (Ultraspec 2000) | Sasseville, Benhaberou-Brun [4] | Safety lenses | 48.6 | 5.4 | 0.14 | 5.6 |
| Hoya | BlueControl | Lin, Gerratt [10] | Task-specific – VDU use | 97.1 | 92.2 | 0.01 | 94.5 |
| JINS CO. LTD | Screen Clear | Website (archived) | Task-specific – VDU use | 94 | 88.6 | 0.03 | 77 |
| JINS CO. LTD | Screen Night | Website (archived) | Task-specific – VDU use | 70.5 | 49.8 | 0.05 | 65.4 |
| LowBlueLights | Blue Light Blocking Glasses-Sleep Glasses | Esaki, Kitajima [13] | Task-specific – Other | 39.5 | 2.3 | 0.16 | 0.4 |
| Multilens | 450 | Plum and Gerull [6], [website](https://web.archive.org/web/20190314200110/http:/satis.de/links/05_downloads/Kantenfilter2008.pdf) | Medical | 72.4 | 46.4 | 0.08 | 25.5 |
| Multilens | 511 | Plum and Gerull [6], [website](https://web.archive.org/web/20190314200110/http:/satis.de/links/05_downloads/Kantenfilter2008.pdf) | Medical | 48.5 | 10.5 | 0.12 | 11.8 |
| Multilens | 527 | Plum and Gerull [6], [website](https://web.archive.org/web/20190314200110/http:/satis.de/links/05_downloads/Kantenfilter2008.pdf) | Medical | 39 | 5.4 | 0.14 | 10.6 |
| Multilens | 550 | Plum and Gerull [6], [website](https://web.archive.org/web/20190314200110/http:/satis.de/links/05_downloads/Kantenfilter2008.pdf) | Medical | 17.2 | 2.5 | 0.21 | 18 |
| Multilens | 450 POL | Plum and Gerull [6], [website](https://web.archive.org/web/20190314200110/http:/satis.de/links/05_downloads/Kantenfilter2008.pdf) | Medical | 25.3 | 14.6 | 0.07 | 45.1 |
| Multilens | 511 POL | Plum and Gerull [6], [website](https://web.archive.org/web/20190314200110/http:/satis.de/links/05_downloads/Kantenfilter2008.pdf) | Medical | 21.1 | 8.1 | 0.09 | 28.5 |
| Multilens | 527 POL | Plum and Gerull [6], [website](https://web.archive.org/web/20190314200110/http:/satis.de/links/05_downloads/Kantenfilter2008.pdf) | Medical | 15.7 | 4.7 | 0.12 | 29.9 |
| Multilens | 550 POL | Hoggan, Subhash [11] | Medical | 8 | 1.6 | 0.19 | 26.9 |
| Multilens | LLR Night Cover | Technical fact sheet, from [website (archived)](https://web.archive.org/web/20190314193744/https:/www.multilens.se/Image/GetDocument/en/44/eng_ml-night-cover.pdf) | Task-specific – Driving | 86.2 | 69.4 | 0.04 | 63.6 |
| NoIR | 47 | [Website (archived)](https://web.archive.org/web/20190314202246/http:/noirmedical.com/465.html) | Medical | 43.5 | 30 | 0.07 | 61.2 |
| NoIR | 50 | [Website (archived)](https://web.archive.org/web/20190314201154/http:/noirmedical.com/47.html) | Task-specific – Sports | 83.6 | 48.4 | 0.09 | 11.2 |
| NoIR | 60 | [Website (archived)](https://web.archive.org/web/20190314201416/http:/noirmedical.com/50uv.html) | Task-specific – Sports | 44.3 | 3.2 | 0.15 | 0.2 |
| NoIR | 68 | [Website (archived)](https://web.archive.org/web/20190314202417/http:/noirmedical.com/505.html) | Task-specific – VDU use | 59.9 | 14.4 | 0.11 | 23.3 |
| NoIR | 72 | [Website (archived)](https://web.archive.org/web/20190314202517/http:/noirmedical.com/533.html) | Medical | 56 | 44.1 | 0.04 | 86.8 |
| NoIR | 90 | [Website (archived)](https://web.archive.org/web/20190314202601/http:/noirmedical.com/553.html) | Medical | 13 | 0.2 | 0.29 | 0.5 |
| NoIR | 93 | [Website (archived)](https://web.archive.org/web/20190314202744/http:/noirmedical.com/570.html) | Medical | 5.6 | 0.2 | 0.28 | 1.5 |
| NoIR | 465 | [Website (archived)](https://web.archive.org/web/20190314201524/https:/noirmedical.com/60uv.html) | Task-specific – Sports | 76.3 | 35.5 | 0.09 | 21.1 |
| NoIR | 505 | [Website (archived)](https://web.archive.org/web/20190314201650/https:/noirmedical.com/68.html) | Task-specific – Sports | 26.4 | 4.1 | 0.13 | 3.6 |
| NoIR | 533 | [Website (archived)](https://web.archive.org/web/20190314201810/http:/noirmedical.com/72.html) | Task-specific – Sports | 22.6 | 7.2 | 0.11 | 43 |
| NoIR | 553 | [Website (archived)](https://web.archive.org/web/20190314202017/https:/noirmedical.com/90uv.html) | Medical | 26.8 | 1.1 | 0.2 | 0.9 |
| NoIR | 570 | [Website (archived)](https://web.archive.org/web/20190314202120/http:/noirmedical.com/93uv.html) | Medical | 12.2 | 0.5 | 0.22 | 2 |
| NoIR | N/A - amber lens | Plum and Gerull [6], [website](https://web.archive.org/web/20190314200110/http:/satis.de/links/05_downloads/Kantenfilter2008.pdf) | Medical | 46.5 | 3.7 | 0.15 | 0.7 |
| Offenhäuser + Berger GmbH | Laserschutzbrille 0000001012726 red | [Website (archived)](https://web.archive.org/web/20190314203748/https:/www.offenhaeuser-berger.de/laserschutzbrillen.php) | Safety lenses | 23.9 | 0.6 | 0.23 | 1.4 |
| Offenhäuser + Berger GmbH | Laserschutzbrille 0000001012731 orange | [Website (archived)](https://web.archive.org/web/20190314203748/https:/www.offenhaeuser-berger.de/laserschutzbrillen.php) | Safety lenses | 7.5 | 0.4 | 0.13 | 0.5 |
| Offenhäuser+ Berger GmbH | Laserschutzbrille 0000003013447 red-orange | [Website (archived)](https://web.archive.org/web/20190314203748/https:/www.offenhaeuser-berger.de/laserschutzbrillen.php) | Safety lenses | 23.2 | 0.7 | 0.19 | 0.6 |
| Offenhäuser+ Berger GmbH | Laserschutzbrille 2054110000002 yellow | Kayumov, Casper [14] | Safety lenses | 58.6 | 9 | 0.12 | 0.2 |
| Offenhäuser+ Berger GmbH | Laserschutzbrille 2054110000002 yellow | [Website (archived)](https://web.archive.org/web/20190314203748/https:/www.offenhaeuser-berger.de/laserschutzbrillen.php) | Safety lenses | 58.3 | 8.9 | 0.12 | 0.4 |
| Rodenstock | L660 | Plum and Gerull [6], [website](https://web.archive.org/web/20190314200110/http:/satis.de/links/05_downloads/Kantenfilter2008.pdf) | Medical | 16.9 | 4.5 | 0.11 | 13.3 |
| Rodenstock | L660 80% | Schwerdtfeger and Gräf [7] | Medical | 15.7 | 3.8 | 0.12 | 20.7 |
| Rodenstock | L660 90% | Burkhart and Phelps [15] | Medical | 9.5 | 2.3 | 0.12 | 23.6 |
| Rosenblum et al 2000 | N/A - filter albino patients | Plum and Gerull [6], [website](https://web.archive.org/web/20190314200110/http:/satis.de/links/05_downloads/Kantenfilter2008.pdf) | Medical | 14.6 | 10.3 | 0.05 | 78.2 |
| Rosenblum et al 2000 | N/A - filter aphakic patients | Rosenblum, Zak [16] | Medical | 86.5 | 73 | 0.03 | 77.3 |
| Rosenblum et al 2000 | N/A - filter initial cataract patients | Rosenblum, Zak [16] | Medical | 75.6 | 47.5 | 0.06 | 51.2 |
| SAF-T-CURE | ORANGE UV FILTER GLASSES | Figueiro and Rea [17] | Safety lenses | 41.1 | 2.7 | 0.16 | 0.6 |
| solsecur / AugenLichtSchutz | red | Zerbini, Kantermann [18] | Task-specific – Other | 42.4 | 12.6 | 0.11 | 33.1 |
| solsecur / AugenLichtSchutz | red xd | [Website (archived)](https://web.archive.org/web/20190314203036/https:/www.augenlichtschutz.de/produkte/augenerkrankungen/over-shield-l-red-xd-breitsicht-schutzbrille) | Safety lenses | 30.5 | 1.2 | 0.2 | 0 |
| solsecur / AugenLichtSchutz | Typ31: P90 | Rosenblum, Zak [16] | Medical | 9 | 4 | 0.1 | 43.1 |
| solsecur / AugenLichtSchutz | yellow sx | [Website (archived)](https://web.archive.org/web/20190314202803/https:/www.augenlichtschutz.de/produkte/sport-freizeit/over-shield-l-yellow-sx-breitsicht-gelbfilterbrille) | Task-specific – Driving | 70.3 | 35.3 | 0.08 | 28.4 |
| SomniLight | Amber Sleep Glasses | Website (archived) | Task-specific – Other | 25.6 | 0.9 | 0.21 | 1.3 |
| SomniLight | Migraine Relief Lenses | Website (archived) | Medical | 5.6 | 0.6 | 0.3 | 8 |
| Swiss Lens | Noflex | Leung, Li [19] | Task-specific – VDU use | 93.5 | 89.8 | 0.01 | 95.2 |
| Swiss Lens | StressFree | Leung, Li [19] | Task-specific – VDU use | 97.2 | 94.8 | 0.01 | 95.9 |
| SwissLens | SLF450 | [Website (archived)](https://web.archive.org/web/20190314205657/https:/www.somnilight.com/photophobia-glasses-and-migraine-glasses.html) | Medical | 83.2 | 46.7 | 0.09 | 16.3 |
| SwissLens | SLF511 | [Website (archived)](https://web.archive.org/web/20190314205657/https:/www.somnilight.com/photophobia-glasses-and-migraine-glasses.html) | Medical | 46.4 | 5.8 | 0.14 | 1.9 |
| SwissLens | SLF527 | [Website (archived)](https://web.archive.org/web/20190314205657/https:/www.somnilight.com/photophobia-glasses-and-migraine-glasses.html) | Medical | 36.1 | 2.8 | 0.17 | 1.7 |
| SwissLens | SLF550 | [Website (archived)](https://web.archive.org/web/20190314205657/https:/www.somnilight.com/photophobia-glasses-and-migraine-glasses.html) | Medical | 20.3 | 0.7 | 0.24 | 2 |
| Ultravision International | Soft contact lenses - orange | Website (archived) | Medical | 55.1 | 31.9 | 0.07 | 70.6 |
| Woehlk | HydroFlexRP | Giménez, Beersma [20] | Medical | 13.4 | 2 | 0.15 | 16.5 |
| Yamamoto Kogaku Co. Ltd | O 360S UV Orange | Esaki, Kitajima [21] | Safety lenses | 40.5 | 2.2 | 0.17 | 0.2 |
| Zeiss | BlueProtect | Leung, Li [19] | Task-specific – VDU use | 99.4 | 96.8 | 0.01 | 95.6 |
| Zeiss | F540 | Plum and Gerull [6], [website](https://web.archive.org/web/20190314200110/http:/satis.de/links/05_downloads/Kantenfilter2008.pdf) | Medical | 50.2 | 12.2 | 0.11 | 11.6 |
| Zeiss | F560 | Schwerdtfeger and Gräf [7] | Medical | 36.7 | 5.5 | 0.14 | 13.3 |
| Zeiss | F580 | Plum and Gerull [6], [website](https://web.archive.org/web/20190314200110/http:/satis.de/links/05_downloads/Kantenfilter2008.pdf) | Medical | 23.3 | 1.5 | 0.22 | 6.1 |
| Zeiss | F580 | Plum and Gerull [6], [website](https://web.archive.org/web/20190314200110/http:/satis.de/links/05_downloads/Kantenfilter2008.pdf) | Medical | 22.9 | 2.9 | 0.19 | 15.4 |
| Zeiss | F60 | Plum and Gerull [6], [website](https://web.archive.org/web/20190314200110/http:/satis.de/links/05_downloads/Kantenfilter2008.pdf) | Medical | 21.3 | 6.1 | 0.11 | 31.5 |
| Zeiss | F80 | Schwerdtfeger and Gräf [7] | Medical | 8.6 | 2.6 | 0.12 | 41 |
| Zeiss | F90 | Plum and Gerull [6], [website](https://web.archive.org/web/20190314200110/http:/satis.de/links/05_downloads/Kantenfilter2008.pdf) | Medical | 4.3 | 2.2 | 0.1 | 67.8 |
| Zeiss | Skylet Fun | Plum and Gerull [6], [website](https://web.archive.org/web/20190314200110/http:/satis.de/links/05_downloads/Kantenfilter2008.pdf) | Task-specific – Other | 27.9 | 10.1 | 0.1 | 45.4 |
| Zeiss | Skylet Road | Plum and Gerull [6], [website](https://web.archive.org/web/20190314200110/http:/satis.de/links/05_downloads/Kantenfilter2008.pdf) | Task-specific – Driving | 19.4 | 11.3 | 0.07 | 55.4 |
| Zeiss | Skylet Sport | Plum and Gerull [6] | Task-specific – Sports | 10 | 5.1 | 0.08 | 59.3 |
| ZELTZER X-CHROM | N/A - final modified red contact lens | Plum and Gerull [6], [website](https://web.archive.org/web/20190314200110/http:/satis.de/links/05_downloads/Kantenfilter2008.pdf) | Medical | 3.7 | 0.1 | 0.27 | 0.7 |
| ZELTZER X-CHROM | N/A - modified red contact lens | Zeltzer [22] | Medical | 8.1 | 0.6 | 0.24 | 11.7 |

**References**

1. BPI, *BPI Therapeutic Tints*. 2006, Miami, FL: BPI.

2. Herz, N.L. and M.T. Yen, *Modulation of sensory photophobia in essential blepharospasm with chromatic lenses.* Ophthalmology, 2005. **112**(12): p. 2208-11.

3. van der Lely, S., et al., *Blue blocker glasses as a countermeasure for alerting effects of evening light-emitting diode screen exposure in male teenagers.* Journal of Adolescent Health, 2015. **56**(1): p. 113–119.

4. Sasseville, A., et al., *Wearing blue-blockers in the morning could improve sleep of workers on a permanent night schedule: a pilot study.* Chronobiology International, 2009. **26**(5): p. 913–925.

5. Sasseville, A. and M. Hébert, *Using blue-green light at night and blue-blockers during the day to improves adaptation to night work: a pilot study.* Progress in Neuro-Psychopharmacology & Biological Psychiatry, 2010. **34**(7): p. 1236–1242.

6. Plum, K. and K. Gerull, *Kantenfilter und seitlicher Blendschutz – ein praktischer Ratgeber*. 2nd ed. 2008, Mainz/Aachen: WVAO, Wissenschaftliche Vereinigung für Augenoptik und Optometrie e.V & Pro Retina Deutschland e.V.

7. Schwerdtfeger, G. and M. Gräf, *Kantenfilterkontaktlinse und Kantenfiltergläser bei Achromatopsie.* Zeitschrift für Praktische Augenheilkunde, 1994. **15**: p. 322-328.

8. Sasseville, A., et al., *Blue blocker glasses impede the capacity of bright light to suppress melatonin production.* Journal of Pineal Research, 2006. **41**(1): p. 73–78.

9. Krüger, J., R. Bullmann, and M. Drechsler, *Ein Selbstversuch: Eine Woche ohne blaues Licht–Auswirkungen auf Aufmerksamkeit, Schlaf und Befinden.* Lux junior, 2017.

10. Lin, J.B., et al., *Short-wavelength light-blocking eyeglasses attenuate symptoms of eye fatigue.* Investigative Ophthalmology & Visual Science, 2017. **58**(1): p. 442–447.

11. Hoggan, R.N., et al., *Thin-film optical notch filter spectacle coatings for the treatment of migraine and photophobia.* Journal of Clinical Neuroscience, 2016. **28**: p. 71–76.

12. Ostrin, L.A., K.S. Abbott, and H.M. Queener, *Attenuation of short wavelengths alters sleep and the ipRGC pupil response.* Ophthalmic and Physiological Optics, 2017. **37**(4): p. 440–450.

13. Esaki, Y., et al., *Wearing blue light-blocking glasses in the evening advances circadian rhythms in the patients with delayed sleep phase disorder: An open-label trial.* Chronobiology International, 2016. **33**(8): p. 1037–1044.

14. Kayumov, L., et al., *Blocking low-wavelength light prevents nocturnal melatonin suppression with no adverse effect on performance during simulated shift work.* Journal of Clinical Endocrinology and Metabolism, 2005. **90**(5): p. 2755–2761.

15. Burkhart, K. and J.R. Phelps, *Amber lenses to block blue light and improve sleep: a randomized trial.* Chronobiology International, 2009. **26**(8): p. 1602–1612.

16. Rosenblum, Y.Z., et al., *Spectral filters in low-vision correction.* Ophthalmic and Physiological Optics, 2000. **20**(4): p. 335-41.

17. Figueiro, M.G. and M.S. Rea, *Lack of short-wavelength light during the school day delays dim light melatonin onset (DLMO) in middle school students.* Neuroendocrinology Letters, 2010. **31**(1): p. 92–96.

18. Zerbini, G., T. Kantermann, and M. Merrow, *Strategies to decrease social jetlag: reducing evening blue light advances sleep and melatonin.* European Journal of Neuroscience, 2018.

19. Leung, T.W., R.W.-H. Li, and C.-S. Kee, *Blue-light filtering spectacle lenses: optical and clinical performances.* PloS one, 2017. **12**(1): p. e0169114.

20. Giménez, M.C., et al., *Effects of a chronic reduction of short-wavelength light input on melatonin and sleep patterns in humans: evidence for adaptation.* Chronobiology International, 2014. **31**(5): p. 690–697.

21. Esaki, Y., et al., *Effect of blue-blocking glasses in major depressive disorder with sleep onset insomnia: A randomized, double-blind, placebo-controlled study.* Chronobiology International, 2017. **34**(6): p. 753–761.

22. Zeltzer, H.I., *Use of modified X-Chrom for relief of light dazzlement and color blindness of a rod monochromat.* Journal of the American Optometric Association, 1979. **50**(7): p. 813–818.
